# Pre-hospital triage performance after standardized trauma courses

**DOI:** 10.1186/s13049-017-0395-8

**Published:** 2017-05-19

**Authors:** Maria Lampi, Johan Junker, Peter Berggren, Carl-Oscar Jonson, Tore Vikström

**Affiliations:** Centre for Teaching & Research in Disaster Medicine and Traumatology, Department of Clinical and Experimental Medicine, Linköping University, SE-58185 Linköping, Sweden

**Keywords:** Mass Casualty Incident, Advanced Trauma Life Support, Pre-Hospital Trauma Life Support

## Abstract

**Background:**

The pre-hospital triage process aims at identifying and prioritizing patients in the need of prompt intervention and/or evacuation. The objective of the present study was to evaluate triage decision skills in a Mass Casualty Incident drill. The study compares two groups of participants in Advanced Trauma Life Support and Pre-Hospital Trauma Life Support courses.

**Methods:**

A questionnaire was used to deal with three components of triage of victims in a Mass Casualty Incident: decision-making; prioritization of 15 hypothetical casualties involved in a bus crash; and prioritization for evacuation. Swedish Advanced Trauma Life Support and Pre-Hospital Trauma Life Support course participants filled in the same triage skills questionnaire just before and after their respective course.

**Results:**

One hundred fifty-three advanced Trauma Life Support course participants were compared to 175 Pre-Hospital Trauma Life Support course participants. The response rates were 90% and 95%, respectively. A significant improvement was found between pre-test and post-test for the Pre-Hospital Trauma Life Support group in regards to decision-making. This difference was only noticeable among the participants who had previously participated in Mass Casualty Incident drills or had experience of a real event (pre-test mean ± standard deviation 2.4 ± 0.68, post-test mean ± standard deviation 2.60 ± 0.59, *P* = 0.04). No improvement was found between pre-test and post-test for either group regarding prioritization of the bus crash casualties or the correct identification of the most injured patients for immediate evacuation.

**Conclusions:**

Neither Advanced Trauma Life Support nor Pre-Hospital Trauma Life Support participants showed general improvement in their tested triage skills. However, participation in Mass Casualty Incident drills or experience of real events prior to the test performed here, were shown to be advantageous for Pre-Hospital Trauma Life Support participants. These courses should be modified in order to assure proper training in triage skills.

## Background

Disaster management is a complex process, involving coordination, medical intervention and effective triage [1, 2]. It is well established that health care professionals must be adequately prepared for a variety of casualty events [3]. The pre-hospital triage process aims at identifying and prioritizing patients in the need of prompt intervention and/or evacuation. Therefore, exercises and training are essential elements for health care personnel in order to be adequately prepared and thus reduce triage errors [4–7]. Although various triage training methods have been developed, validation so far is fragmentary [8]. A simple instrument that is easy to remember, easy to use, and has the ability to evaluate the injured patient is needed [9]. Several national and international triage systems exist to support providers in complex triage decisions, for example Simple Triage And Rapid Treatment (START) [10] and Sort, Assess, Life-saving intervention treatment, and/or transport Triage system (SALT) [8]. Still, no common international guidelines for mass casualty triage exist. The absence of guidelines has resulted in variability in the triage processes, the use of tags and nomenclature. There is also limited evidence of the validity of existing triage tools [11, 12].

Physicians involved in the initial patient treatment in a disaster situation could play a key role if they are properly prepared and trained in Advanced Trauma Life Support (ATLS) [13, 14]. Furthermore, previous studies have indicated that the experience of physicians or pre-hospital personnel who have undergone training and who work daily in a pre-hospital setting can be of benefit during a Mass Casualty Incident (MCI) [4, 15, 16].

According to the Swedish emergency response system, in case of a MCI, the first ambulance on scene is typically crewed with one Emergency Medical Technician (EMT) and one specialist registered nurse. The registered nurse will be appointed medical incident commander and normally retain this position for the duration of the incident. If a physician arrives to the incident site, he or she focuses on acute medical assessment and acts as an advisor on medical decisions. In Sweden, which professional that should be the medical incident commander is under debate. No clear consensus has been reached as of now. The Rescue Services Incident Commander is responsible for rescue and safety [17].

The Advanced Trauma Life Support (ATLS) guidelines have been accepted worldwide as a training concept for medical doctors [18, 19]. Swedish physicians normally attend an ATLS-course during residency. The objective of the ATLS course is to provide participants with skills to identify and treat life-threatening injuries. During the course, trauma triage principles are introduced and training is given by applying the mnemonic ABCDE [20]. A parallel course is the Pre-Hospital Trauma Life Support (PHTLS), accepted worldwide and developed by the American College of Surgeons. PHTLS is based on the ATLS program. However, the PHTLS course is conducted for pre-hospital personnel with focus on pre-hospital trauma care. The PHTLS course objective is to influence critical thinking together with appropriate knowledge and skills to emphasis optimal pre-hospital trauma management [21]. In Sweden, the course is generally attended during the first period of employment as an EMT or registered nurse. The ATLS and PHTLS courses are designed to teach providers a standardized approach to trauma assessment and the sequences combine the educational formats of lectures and practical lifesaving skills. The ATLS course focuses on in-hospital assessment and treatment, whereas the PHTLS course targets pre-hospital care. The triage group discussion within the ATLS course is aimed at training the participants in pre-hospital triage. In the ATLS course, a group discussion based on specific scenarios aims at teaching skills in assessment and triage in a pre-hospital setting, some which are applicable during a mass casualty incident. The PHTLS manual includes the division “Mass Casualties and Terrorism”, which contains elements of disaster management and mass casualty incident management”. Both courses emphasize that injury kills within a certain time frame and evaluation and interventions should follow the structured examination from A to E [20, 21].

The aim of effective emergency medical response is to use accessible resources in the most efficient way and make decisions to reduce the mortality of critically injured patients. It is stated in the ATLS manual: “*it is vital that the ATLS-provider have a working understanding of the applications of ATLS-principles in disaster situations*” [20]. There is a need to establish whether the ABCDE algorithm used in current ATLS and PHTLS courses is sufficient to perform triage.

The hypothesis of the present work is that current ATLS and PHTLS curricula are sufficient for triage in a simulated major incident. This was evaluated using written tests before and after attending respective courses [22, 23].

## Methods

The study was designed as a prospective cross-sectional survey. An instrument that has been developed by Deluhery et al. [22] translated to Swedish and adapted to meet Swedish conditions by Lampi et al. [24] was chosen for the study. The data were collected from two separate groups, an ATLS group and a PHTLS group, during two different periods. Data regarding the ATLS group was collected during a previously performed and published study [24]. Here, it is used in context of comparison to the data gathered from the PHTLS course participants.

The first group included 169 students from the ATLS provider course from ten course sites in Sweden during spring 2012. The second group included 181 students from the PHTLS provider course from nine course sites in Sweden during spring 2013. Demographic data collection included the level of medical education, the number of years in clinical practice, previous experience of simulation exercises and previous experience of real incidents with more than five injured. In some Swedish counties, triage tags should be used in pre-hospital settings where more than five casualties are involved in an incident, independently of the severity of injuries [25].

Triage skills questionnaires were delivered with written instructions to the local ATLS and PHTLS faculties at the course sites. The coordinators, one at each local faculty, were also briefed about the study by phone. The local instructors distributed the triage skills questionnaire to the participants. Together with the pre-course test, the participant was given a written information letter explaining the aim, goals and information about anonymity and voluntary participation in the survey. All participants gave informed consent.

The ATLS and PHTLS course participants filled in the same triage skills questionnaire just before and after the course. The questionnaire was divided into three sections. The first section contained three multiple-choice questions, each with three alternatives, focusing on different levels of decision-making in a mass casualty incident. A maximum of three points, one per correct answer could be obtained. This score was used as a measure of the participants’ decision-making ability. The second section involved an assignment to triage 15 hypothetical casualties involved in a bus crash. Each patient was presented with a description contained age, sex, visible injuries and information regarding quality and rate of pulse and breathing. Example of one patient description (author’s translation): *Doesn’t wave or move when instructed - 36-year-old female with a penetrating shrapnel wound to the head that goes through and through. The patient is unresponsive, has shallow respirations approximately two per minute, and no palpable radial pulse*. The participants were informed that they were alone at the scene and would be for some time, and more resources had been alerted. The participants’ task was to prioritize the 15 casualties according to the ABCDE algorithm and take into account the postulated circumstances. A colour-coded algorithm, red for priority 1 (*n* = 3), yellow for priority 2 (*n* = 3), green for priority 3 (*n* = 7) and black for dead (*n* = 2) was used, in accordance to the ABCDE and SALT triage algorithms [20, 26, 27]. A maximum of 15 points could be obtained, one point per correctly triaged patient. This score was used as a measure of the participants’ triage skills. To validate the triage outcome of the hypothetical casualties, several triage algorithms (Simple Triage and Rapid Treatment, Triage Sieve, CareFlight) have been employed, all yielding the same results.

The third and last section in the questionnaire was related to evacuation from the scene. This task was designed to confirm the triage skills. The participants were informed that there were three ambulances available for evacuation. Their assignment was to identify the three casualties who were the most injured patients that should leave the scene in these three ambulances immediately and at the same time. The participant either managed to identify all three casualties correctly (yes) or failed (no).

The time for completing the triage skills questionnaire in both settings was 15 min. The participants were asked to code the pre- and post-tests for matching. After pairing the questionnaires, the local instructors decoded the questionnaire and sent them by mail for analysis.

The data from the questionnaires were coded, collected and stored in accordance with County Council and University integrity protocols. The regional ethics board was consulted and agreed that the study was not subject to ethical board regulation.

The collected data were recorded in Excel 2010 (Microsoft,14.0.7173.5000,SP2) for MAC. SPSS Release 23.0.0.0 (IBM, Ref 4040559) and Prism 6.0 (GraphPad, LaJolla, US) were used for statistical analyses. Kolmogorov-Smirnov tests were used to assess normal distribution of study groups. To test for significant differences between groups in the first and second section of the questionnaire, Kruskal-Wallis tests coupled with Dunn´s multiple comparisons tests were used. Results from the last section of the questionnaire were compared using chi-squared tests. A *P* value of less than 0.05 was considered statistically significant.

## Results

A total of 350 participants (PHTLS, *n* = 181; ATLS, *n* = 169) were included in this survey. A total of 153 (90%) from the ATLS group and 175 (96%) participants from the PHTLS group participated in the survey. All ATLS participants were physicians: 42 interns, 94 residents, 4 attending physicians, 5 senior attending physicians and 8 others. PHTLS participants included 12 EMTs, 89 registered nurses, 54 specialist nurses, 5 physicians and 4 other specialists. Sixteen students in the ATLS group and six in the PHTLS group chose not to participate in the survey.

The first section of the triage skill questionnaire contained three multiple-choice questions related to triage decision-making in a mass casualty event. The participants could obtain a maximum score of three points in this section. For the ATLS participants, pre-test scores were 2.58 ± 0.55 (mean ± standard deviation), and post-test scores were 2.65 ± 0.55. This difference was not statistically significant (*P* = 0.636). Moreover, no significant difference could be observed when only including ATLS participants with prior experience in MCI drills or real events (pre-test: 2.50 ± 0.59, post-test: 2.66 ± 0.57, *P* = 0.371) (Table 1).

**Table 1:** Results from section one in the questionnaire: decision-making

	*n*	Pre-test score Mean ± SD	Post-test score Mean ± SD	Mean Rank difference	*P*	H(df)
ATLS						
Whole group	153	2.58 ± 0.55	2.65 ± 0.55	−26.35	0.64	18.92 (3)
With MCI experience	62	2.5 ± 0.59	2.66 ± 0.57	−49.44	0.37	23.05 (3)
PHTLS						
Whole group	175	2.34 ± 0.74	2.57 ± 0.61	−54.05	0.008	18.92 (3)
With MCI experience	130	2.4 ± 0.68	2.60 ± 0.59	−51.77	0.04	23.05 (3)

A Kruskal-Wallis test with a Dunn’s Multiple Comparisons test was used to test for significant differences between the groups

*n* number of samples, *SD* Standard Deviation, *H* Kruskal-Wallis statistic, *df* degrees of freedom

Scores for the PHTLS participants significantly increased from 2.34 ± 0.74 in the pre-test, to 2.57 ± 0.61 in the post-test (*P* = 0.008). PHTLS participants with prior experience from MCI drills or real events increased their score from 2.4 ± 0.68 pre-test to 2.6 ± 0.59 post-test. This difference was also statistically significant (*P* = 0.04) (Table 1).

In the second section of the questionnaire, the students could obtain a score of 15 patient points, one point for each correctly triaged patient. For the ATLS participants, pre-test scores were 9.46 ± 1.48, and post-test scores were 9.19 ± 1.68. This difference was not statistically significant (P > 0.999). Moreover, no significant difference could be observed when only including ATLS participants with prior experience in MCI drills or real events (pre-test: 9.44 ± 1.51, post-test: 9.26 ± 1.70, *P* = 0.967) (Table 2).

**Table 2:** Results from section two in the questionnaire: points

	*n*	Pre-test score Mean ± SD	Post-test score Mean ± SD	Mean Rank difference	*P*	H(df)
ATLS						
Whole group	153	9.46 ± 1.48	9.19 ± 1.68	23.12	>0.999	3.22 (3)
With MCI experience	62	9.44 ± 1.50	9.26 ± 1.70	9.82	>0.999	7.18 (3)
PHTLS						
Whole group	174	9.51 ± 1.78	9.48 ± 1.62	1.22	>0.999	3.22 (3)
With MCI experience	130	9.62 ± 1.74	9.56 ± 1.54	2.58	>0.999	7.18 (3)

A Kruskal-Wallis test with a Dunn’s Multiple Comparisons test was used to test for significant differences between the groups

*n* number of samples, *SD* Standard Deviation, *H* Kruskal-Wallis statistic, *df* degrees of freedom

Scores for the PHTLS participants were 9.51 ± 1.78 in the pre-test, to 9.48 ± 1.62 in the post-test. This difference was not statistically significant (*P* > 0.999). PHTLS participants with prior experience from MCI drills or real events changed their score from 9.62 ± 1.74 pre-test to 9.59 ± 1.54 post-test. This difference was not statistically significant (*P* > 0.999) (Table 2).

The last section of the questionnaire included a question regarding prioritizing and evacuation of the casualties from the scene. This question was separate from the triage section. The results indicated whether the students had identified the most critical patients. The delta between post and pre values for the prioritisation task was calculated and used as input for a Chi-square analysis where the ATLS group was compared to the PHTLS group. A negative value indicated negative change (better before than after). A zero value indicated no change, whereas a positive value indicated positive change. No significant differences were found (*χ* ^2^ = 2.201, df = 2, *p* = 0.333) (Table 3).

**Table 3:** Descriptive data for section three in the questionnaire: prioritisation

	Negative change	No change	Positive change
ATLS	*N* = 8	*N* = 134	*N* = 5
PHTLS	*N* = 15	*N* = 147	*N* = 3

## Discussion

Triage in the pre-hospital setting is influenced by many different considerations that are well known and documented [7]. One of the challenges in a MCI is to identify the most severely injured, performing life-saving initial medical interventions, and achieving evacuation without unnecessary delay to the correct facility. In the present study, triage skills of participants attending ATLS or PHTLS courses were compared in a simulated MCI using a modified version of the Deluhery et al. questionnaire [22, 24]. Prior to attending the respective courses, ATLS participants scored significantly higher than PHTLS participants in the part of the questionnaire related to decision-making. While the PHTLS participants significantly increased their score after the course, ATLS participants did not. No significant difference was found when comparing the two groups after the respective courses. The significant increase of scores of the PHTLS group was largely due to participants with previous MCI experience. Thus, previous experience of MCI drills or real events is a contributing factor in acquiring triage skills for PHTLS course participants.

In section two of the questionnaire, the triage skills were measured. The average scores of all participants regardless of course attended were only 9 of the possible 15 points, with no significant difference between groups. This must be considered as unexpectedly low. Furthermore, there were no observed effects of scores following participation in either of the courses.

The last section of the questionnaire evaluated the participants’ ability to identify the most critical casualties. No differences were observed between any of the groups, further revealing a deficit in MCI management of the course participants.

The outcome of this study indicates that ATLS and PHTLS participants do not have enough triage preparedness to make sufficient and proper assessment including triage and first priority for evacuation in a simulated MCI. The results gathered in the present study were obtained using a questionnaire regarding a simulated MCI, and thus the transferability of the results to a real context may be limited. However, if translated to a real event, the lack of triage skills of the participants would be a cause of concern. The apparent threat of a real MCI occurring is evident, thus requiring full execution of the Swedish medical disaster response system, which would include many of the physicians and nurses who took part in this study. The efficacy of their real-life triage performance should further be evaluated and related to patient outcome.

In this study, a mnemonic algorithm, well known and used in the hospital setting is used in a pre-hospital setting. The ATLS manual states that “*Triage decisions are typically made by deciding which patients´ injuries constitute the greatest immediate threat of life. As such, the Airway, Breathing, Circulation and Disability priorities of ATLS are the same priorities used to make triage decisions*” [20]. The results obtained in this study indicate that the PHTLS participants significantly improves their ability to make decisions in a MCI, whereas ATLS participants did not. This is surprising, since the ATLS course contains a compulsory group discussion where the aim is to manage multiple patient scenarios and apply trauma triage principles using the mnemonic ABCDE. The PHTLS course had no such requirement or triage lecture or equivalent in the course content. However, the PHTLS manual contains a chapter including triage and initial stabilization.

In the Swedish pre-hospital medical command and control system, a specialized trained nurse is appointed as a medical incident commander. It has been frequently discussed what kind of medical profession the medical incident commander should have. To our knowledge, a comparison regarding triage skills between physicians and ambulance personnel has not been sufficiently studied. Using data acquired from a previous study [24] allowed for this type of comparison between physicians (ATLS) and ambulance personnel (PHTLS). In this study, higher formal medical education did not seem to be of significant advantage for acquiring triage skills. Real experience, opportunity for training and familiarity with the pre-hospital environment may be essential components that can serve as a solid basis for making more accurate triage decisions in an MCI [4, 16].

## Conclusion

Current ATLS and PHTLS curricula might not be sufficient to train medical personnel in triage performance. ATLS and PHTLS participants did not improve general triage skills as assessed in a simulated MCI. However, PHTLS participants were found to significantly increase their skills related to decision-making following course participation. Taken together, the results from this study illustrate a general lack of triage skills necessary to correctly manage a MCI. The ATLS and PHTLS courses should be modified to ensure that participants are properly trained in triage. Alternatively, specific training in triage should be mandated for medical personnel potentially responding to MCIs.

## Abbreviations

ATLS: Advanced Trauma Life Support
EMT: Emergency Medical Technician
MCI: Mass casualty incident
PHTLS: Pre-hospital trauma life support
SALT: Sort, Assess, Life-saving intervention treatment, and/or transport triage system
START: Simple Triage And Rapid Treatment
